# AI-Assisted Forward Modeling of Biological Structures

**DOI:** 10.3389/fcell.2019.00279

**Published:** 2019-11-14

**Authors:** Josh Lawrimore, Ayush Doshi, Benjamin Walker, Kerry Bloom

**Affiliations:** ^1^Department of Biology, The University of North Carolina at Chapel Hill, Chapel Hill, NC, United States; ^2^Department of Mathematics, The University of North Carolina at Chapel Hill, Chapel Hill, NC, United States

**Keywords:** kinetochore, machine learning, convolutional neural network, feature extraction, forward modeling

## Abstract

The rise of machine learning and deep learning technologies have allowed researchers to automate image classification. We describe a method that incorporates automated image classification and principal component analysis to evaluate computational models of biological structures. We use a computational model of the kinetochore to demonstrate our artificial-intelligence (AI)-assisted modeling method. The kinetochore is a large protein complex that connects chromosomes to the mitotic spindle to facilitate proper cell division. The kinetochore can be divided into two regions: the inner kinetochore, including proteins that interact with DNA; and the outer kinetochore, comprised of microtubule-binding proteins. These two kinetochore regions have been shown to have different distributions during metaphase in live budding yeast and therefore act as a test case for our forward modeling technique. We find that a simple convolutional neural net (CNN) can correctly classify fluorescent images of inner and outer kinetochore proteins and show a CNN trained on simulated, fluorescent images can detect difference in experimental images. A polymer model of the ribosomal DNA locus serves as a second test for the method. The nucleolus surrounds the ribosomal DNA locus and appears amorphous in live-cell, fluorescent microscopy experiments in budding yeast, making detection of morphological changes challenging. We show a simple CNN can detect subtle differences in simulated images of the ribosomal DNA locus, demonstrating our CNN-based classification technique can be used on a variety of biological structures.

## Introduction

**FIGURE 1:**
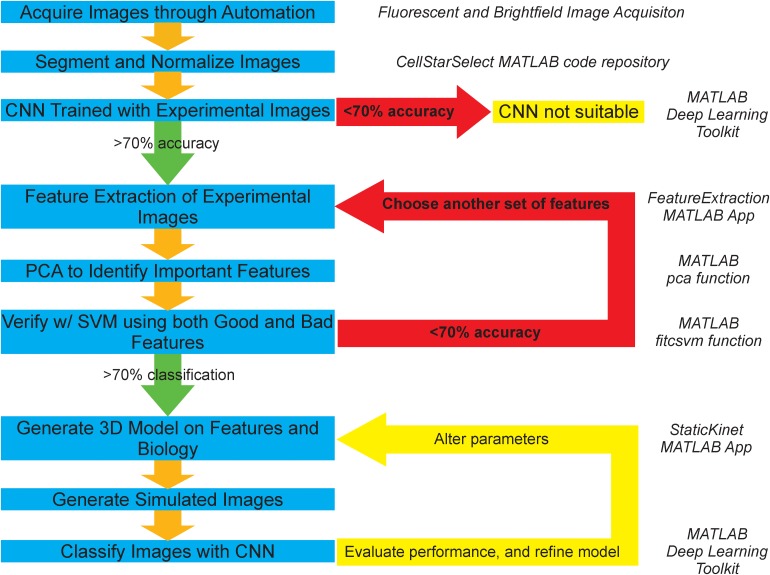
Graphical representation of AI-assisted, forward modeling method. Blue boxes denote steps in the pipeline and yellow boxes denote termination or iteration points. Green arrows depict a successful validation/classification and progression to the next step, while red arrows depict failed validation/classification and repetition of previous steps. A convolutional neural network (CNN) is a deep learning classification algorithm that learns distinguishing features directly from the images. A support vector machine (SVM) is a machine learning classification algorithm that learns from a given *n*-dimensional dataset of image features calculated from the images. Principal component analysis (PCA) is a statistical procedure that calculates vectors that are linear combination of the original features. The new vectors are calculated to generate the maximum amount of variance from the dataset. Each vector is ranked by the amount of variance in the transformed dataset. The importance of the original features is determined by the amount of the original feature in a vector multiplied by the amount of variance of the new vector in the transformed dataset. Italicized text on the right of the figure indicates the program associated with a given step.

Forward modeling relies on the construction of an accurate simulation of a biological structure. These models are built using prior knowledge and can be compared to experimental data to either validate the model or simulate alterations to the structure. Budding yeast has proven to be a useful model of macromolecular models given the ease of fluorescent labeling of proteins and live cell imaging. Previous studies have generated models of microtubule dynamics, centromeric DNA, and the entire genome using budding yeast (Gardner et al., 2008; Wong et al., 2012; Stephens et al., 2013a, b; Lawrimore et al., 2016; Hult et al., 2017; Walker et al., 2019). However, proper assessment of a model’s accuracy is complex and laborious. Here, we describe a method that incorporates computational modeling, simulated image generation, deep learning, principal component analysis, and machine learning to aid in the generation and assessment of computational models. Previous fluorescent microscopy studies utilized deep learning, reviewed here (LeCun et al., 2015; Kraus et al., 2016; Pärnamaa and Parts, 2017; Sullivan et al., 2018; Lu et al., 2019). The method described herein trains a CNN on simulated fluorescent images from computational models programed to simulate the parameters deemed important by principal-component-analysis (PCA)-based exploratory data analysis (Figure 1) or the user. Experimental images are then classified by the CNN as a computational model. Therefore, our method utilizes CNN classification to determine which computational model best matches a given experimental image. We chose to utilize models of the budding yeast kinetochore and ribosomal rDNA locus (rDNA), but any biological structure that can be digitally imaged and spatially modeled by computer simulation can utilize this method.

The kinetochore, composed of more than 100 proteins (Biggins, 2013), is a cylindrical-shaped distribution of proteins assembled at centromeres of chromatids in eukaryotic cells and links the centromere to microtubule plus-ends (Joglekar et al., 2009; Musacchio and Desai, 2017). The kinetochore complex also plays an important role in the spindle assembly checkpoint by confirming that all the chromosomes are attached to the spindle in a bipolar orientation prior to the separation of the sister chromatids (Lara-Gonzalez et al., 2012; Joglekar, 2016; Joglekar and Kukreja, 2017). The kinetochore complex can be separated into two regions: the inner and outer kinetochore (Cheeseman et al., 2006; Fukagawa and Earnshaw, 2014; Huis in ’t Veld et al., 2016; Musacchio and Desai, 2017). The inner kinetochore is composed of proteins that are localized close to the chromatin, interacting with the DNA. The proteins that are part of the inner kinetochore in *S. cerevisiae* include Cse4, Ame1, and Okp1 (Stoler et al., 1995; Pot et al., 2005; Hornung et al., 2014; Schmitzberger et al., 2017; Anedchenko et al., 2019). The outer kinetochore is composed of proteins that are localized close to microtubules and contain the microtubule-binding domain of the kinetochore complex. The proteins that are part of the outer kinetochore in *S. cerevisiae* include Nuf2, Ndc80, and Spc24 (He et al., 2001; Alushin and Nogales, 2011; Lampert and Westermann, 2011).

The budding yeast *S. cerevisiae* is a useful model organism for the kinetochore complex. Several of the proteins in the kinetochore complex of *S. cerevisiae* have direct homologs to other eukaryotic organisms, most importantly humans, allowing for understandings in *S. cerevisiae* to be translated to humans with relative ease (Kitagawa and Hieter, 2001). Furthermore, a large body of work on the genetics and nuclear dynamics of *S. cerevisiae* already exists, providing a useful foundation that can be further expounded upon. Lastly, while the general structure of the kinetochore complex of *S. cerevisiae* is similar to that of other eukaryotic organisms, simplifications in the structure allow for easier modeling and analysis, such as the one-to-one binding nature of a kinetochore to a microtubule that is not present in higher eukaryotic organisms (Peterson and Ris, 1976). Much work on understanding the kinetochore in the *S. cerevisiae* model has been previously performed, which include analyzing the 3D structure through electron microscopy, characterizing key kinetochore protein interactions, and identifying systematic differences between inner and outer kinetochore proteins (Peterson and Ris, 1976; Stoler et al., 1995; Pot et al., 2005; Lampert and Westermann, 2011; Haase et al., 2012; Huis in ’t Veld et al., 2016; Joglekar and Kukreja, 2017; Musacchio and Desai, 2017).

Unlike the kinetochore, the nucleolus, the nuclear body that surrounds the ribosomal DNA locus in the budding yeast genome, appears as an amorphous crescent adjacent to the nuclear envelope in budding yeast during G1 of the cell cycle (Yang et al., 1989; Léger-Silvestre et al., 1999). Previous studies have modeled the nucleolus as a region of the genome enriched in DNA crosslinking that compacts the rDNA locus into several distinct clusters. Altering the kinetics of the crosslinks in the model changes the properties of the clusters and simulated fluorescent images of the model’s rDNA locus (Hult et al., 2017; Walker et al., 2019). Given the difficulty in detecting morphological changes in the rDNA locus/nucleolus in fluorescence images, we sought to determine if a simple CNN could correctly classify simulated fluorescence images generated from models with different DNA crosslinking kinetics.

The process of acquisition, segmentation, and analysis of microscopy images to gain insight into biological structures is limited to an inefficient and slow binary comparison of features susceptible to human bias, and does not provide any avenue for future classification of phenotypes or structures without spending large amounts of time and resources to train an individual to do so by eye. To address these concerns and provide a method that is more objective, resourceful, and time-efficient. We propose a novel pipeline (Figure 1), which is based on publicly available segmentation algorithms, deep learning and machine learning techniques, and basic statistical procedures (Versari et al., 2017).

The experimental branch of the pipeline begins by automatically detecting and segmenting budding yeast undergoing mitosis. The resulting images are then processed to remove noise and background fluorescence. An initial test on whether the physical features of the two conditions differ is then run by using a CNN. If the neural network fails to successfully both train and categorize the two conditions correctly at an accuracy of 70% or higher, then the two conditions are deemed to appear too similar for this analysis pipeline. However, if the neural network does successfully both train and categorize the two conditions, then the features that are thought to be important are extracted from the two sets of images. Principal component analysis is then used to identify the features that are of greatest importance to the distribution. These features are then validated through successful segregation of the two distributions using a support vector machine (SVM). If the SVM fails to correctly categorize the two conditions at an accuracy greater than 70% based on the features shown to be important, or if support vector machines trained on important and unimportant features have equal accuracy, then additional features are chosen and the process of extraction, identification, and validation of important features is repeated. However, if the validation is successful and only the important features build a valid segregating hyperplane, those features are then used as the basis for the development of a 3D model that can output simulated microscope images. The accuracy of the 3D model is then explored through successful classification of the simulated images by a CNN that is trained on experimental images or the classification of experimental images by a CNN that is trained on simulated images. This procedure of building a model and analyzing it is repeated to develop and validate computational models of biological structures.

To test the pipeline’s effectiveness in discerning differences in physical characteristics, we used a test case comparing the inner kinetochore, represented by Cse4, and outer kinetochore, represented by Nuf2. A simple CNN was able to distinguish experimental images of fluorescently tagged Cse4 and Nuf2. Two known metrics differentiating Nuf2 from Cse4 in mitotic yeast is the width of their distributions perpendicular to the spindle (Haase et al., 2012) and their distance from the spindle pole body (SPB), the microtubule organizing centers in yeast (Haase et al., 2013). These metrics would allow us to determine if PCA would determine these metrics as important. Indeed, these two metrics were found to be important in our PCA-based, exploratory data analysis. Lastly, we trained a CNN on simulated images of fluorescently tagged Cse4 that varied in a single model parameter, the width of Cse4 distribution. We then used the trained CNN to predict the width of the Cse4 distribution from experimental images.

To test if our method could be applied to amorphous biological structures, we tested if a CNN could distinguish simulated images from polymer models of the budding yeast nucleolus. A previous study had shown that the simulated fluorescent signal of the nucleolus increased in area, decreased in standard deviation, and exhibited fewer local maxima as the crosslinking rate increased (Walker et al., 2019), indicating the simulated, nucleolar images should be visually distinct. Indeed, our CNN was able to distinguish between simulated images created using different model parameters, demonstrating the extensibility of our method.

## Materials and Methods

### Experimental Image Acquisition, Segmentation, and Normalization

Budding yeast strain YEF 473A was transformed with SPC29-RFP:HYG^R^ to fluorescently label the SPBs to generate the strain KBY7999. Strain KBY7999 was transformed with GFP-NUF2:NAT^R^ to generate strain KBY8169. Budding yeast strain YEF 473A was transformed with pKK1 to fluorescently label Cse4 with GFP and the endogenous Cse4 was removed and replaced with HYG^R^ to generate strain KBY2010. Strain KBY2010 was transformed with SPC29-RFP:KAN^R^ to fluorescently label the SPBs and generate strain DCY1196.1. Seven Z-plane image stacks of Spc29-RFP, N-terminal GFP-Nuf2 (KBY8169) and Spc29-RFP, Cse4-GFP (DCY1196.1) yeast strains were acquired with a Nikon Eclipse Ti TE2000-U inverted fluorescent microscope using a Nikon Apo 1.4 NA 100x objective, MetaMorph 7.8 software, Hamamatsu Orca Flash 4.0 LT camera, and LumenCor Aura Light Engine. The cells in the images were segmented using a MATLAB code repository, CellStarSelect^1^, that utilizes the CellStar segmentation algorithm (Versari et al., 2017) for segmenting budding yeast buds from brightfield microscopy images. The MATLAB function spotDetection calls the CellStar program to segment yeast buds and uses the function advPointSourceDetection.m^2^ (Cicconet et al., 2017), which was based code developed for Aguet et al. (2013), to detect kinetochore and SPB foci within the bud segment in the fluorescent image channels. If two kinetochore foci and two SPB foci are detected, the bud segment is analyzed and saved in a cell array. The function compileImages.m parses the cell array containing the segmented fluorescent images and creates a 50 × 50-pixel image around each bud that contained two kinetochore and SPB foci. The seven-step 50 × 50-pixel stack of each bud was condensed into a single plane using a maximum projection approach and had their intensity values normalized and saved as 16-bit, RGB images. Duplicates of the images were generated by rotating the images to generate seven additional orientations of the initial to increase the size of the dataset. The images then underwent a background subtraction procedure and were de-noised using a low-pass 2D Wiener filter. All image processing codes are contained within the CellStarSelect repository^1^.

### Training and Testing of a Convolutional Neural Network

We generated a CNN using MATLAB’s Deep Learning Toolbox (Mathworks, Natick, MA, United States). The following summarizes the code from the MATLAB script CNNBasic.m^3^. Image sets of the categories of interest were randomized and split into training, validation, and testing data stores, with 56% of the images used for training, 24% used for validation, and 20% used for testing. The architecture of the CNN contained 13 layers with learn-able weights. The first 12 layers consisted of a 4-layer pattern repeated three times, with the first layer consisting of a 3 × 3 convolutional layer with a stride of 1 and 8, 16, and 32 filters, respectively. The second layer was a batch normalization layer, the third layer was a rectified linear unit layer, and the fourth layer was a 2 × 2 max-pooling layer with a stride of 2. However, on the final repetition of the four layers, the fourth layer was a fully connected layer, with an output size set to the number of classification categories, that fed into a SoftMax layer. The training used stochastic gradient descent with momentum as well as the associated default values for this method in MATLAB, with the exception of the initial learning rate set to 0.01, the max epochs set to 20, and a validation frequency set to 30 iterations.

### Feature Extraction and Principal-Component-Analysis-Based Feature Evaluation

Kinetochore features were extracted from the rescaled, 16-bit, RGB, experimental and simulated images using the FeatureExtraction.mlapp MATLAB application^4^. The following features were extracted from the images: spot height (width of foci perpendicular to the spindle) of SPB (SPC29-RFP) foci and kinetochore (Cse4-GFP or GFP-Nuf2), the standard deviation of linescans of SPB and kinetochore foci parallel (X), perpendicular (Y) to the spindle, and of a 5 × 5 cropped segment of the foci, the mean intensity of a 5 × 5 cropped segment of the SPB and kinetochore foci, the distance of the two kinetochore foci, the distance of the kinetochore foci to the proximal SPB foci, and the distance of the kinetochore foci to the proximal SPB foci parallel to the spindle (the *X*-axis component of the kinetochore to SPB distance).

Features of interest (Table 1) from all classification categories were combined, normalized using MATLAB’s built-in normalize.m function, and subjected to principal component analysis. Principal component analysis was performed with MATLAB’s built-in pca.m function. The pca.m function outputted the principal components, stored in the coeff matrix variable, and the associated percentage of the total variance explained by each principle component, stored in the explained array variable. The importance of the features was determined by multiplying the square of each of the principal components by the percentage of the variance that principal component explained, and the resultant values were ranked in descending order. These analyses were performed by PCAFeatureExtraction.m for comparing data from experimental images, PCASimFeatureExtraction.m for comparing data from simulated images of inner kinetochore models, and PCASimInnerOuterFeatureExtraction.m for comparing data from simulated images of inner and outer kinetochore proteins. These functions are available at https://github.com/BloomLabYeast/FeatureExtractionAndSVM.

**TABLE 1:** Features of interest ranked in order of their importance by PCA.

**Feature**	**Rank**	**Description**
Kinetochore distance to SPB	1	Euclidean distance between the brightest pixel of the kinetochore and the brightest pixel of its closest SPB foci normalized by the length of the spindle
Kinetochore X-distance	2	Magnitude of the distance between the brightest pixel of the kinetochore and the brightest pixel of its closest SPB foci parallel to the spindle axis, normalized by the length of the spindle
SPB foci mean intensity	3	Mean intensity of a 5 × 5 region encompassing the SPB foci
SPB foci height	4	Full-width-half-max of the maximum projection of the 7 × 15 region about the brightest pixel of the SPB foci perpendicular to the spindle
SPB foci STD in Y	5	Standard deviation of the intensities generated from a line-scan of a SPB foci perpendicular to the spindle
SBP foci STD in X	6	Standard deviation of the intensities generated from a line-scan of a SPB foci parallel to the spindle
SPB Foci STD	7	Standard deviation of intensities of 5 × 5 region encompassing the SPB foci
Kinetochore foci height	8	Full-width-half-max of the maximum projection of the 7 × 15 region about the brightest pixel of the kinetochore foci perpendicular to the spindle
Kinetochore foci STD	9	Standard deviation of intensities of 5 × 5 region encompassing the kinetochore foci
Kinetochore foci STD in Y	10	Standard deviation of the intensities generated from a line-scan of a SPB foci parallel to the spindle
K–K distance	11	Euclidean distance between the brightest pixels of the two kinetochore foci, normalized by the length of the spindle axis
Kinetochore foci mean intensity	12	Mean intensity of a 5 × 5 region encompassing the kinetochore foci
Kinetochore foci STD in X	13	Standard deviation of the intensities generated from a line-scan of a kinetochore foci parallel to the spindle

### Training and Testing Binary Gaussian Kernel

Observations and associated data from the feature extraction were randomized and split into training and testing datasets, with 70% of the images used for training and the remaining 30% used for testing. The training set was then used to train a binary Gaussian kernel-based classification model using a MATLAB program, runSVM.m (see text footnote 5). The classifier used a support-vector-machine-based response range and a deviance loss function with a variable regularization term strength, kernel scale parameter, and the number of dimensions of expanded space. The testing set was then classified by the classifier and the accuracy calculated.

### 3D Modeling and Simulated Imaging of the Budding Yeast Kinetochore

**FIGURE 2:**
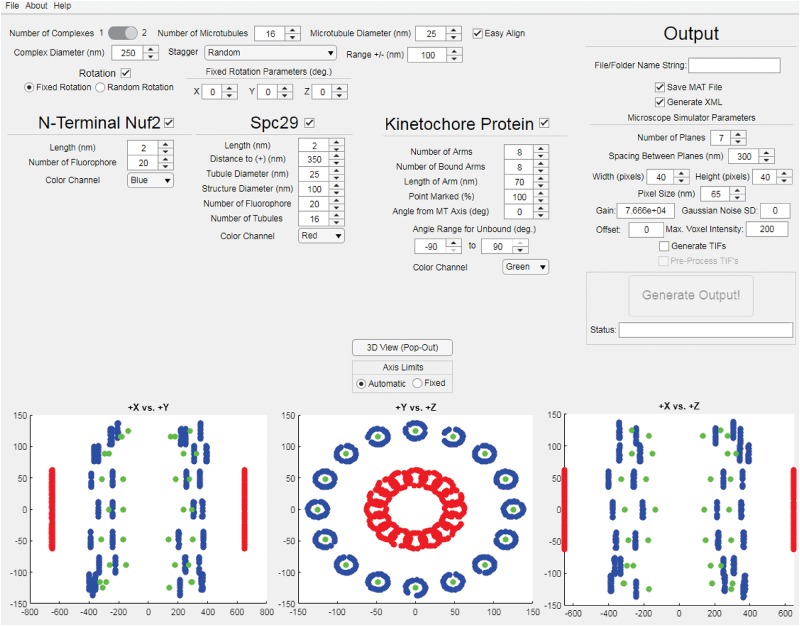
Kinetochore Model Graphical User Interface. The interface of the StaticKinet MATLAB application used to generate simulated fluorescent images of kinetochore models. In the bottom three panels the red dots represent fluorophores bound to the minus ends of the kinetochore microtubules (to approximate the spindle pole bodies), the blue dots represent the N-termini of Nuf2 bound to the plus ends of the kinetochore microtubule, and the green dots represent a given kinetochore protein, i.e., Cse4.

The StaticKinet MATLAB application (Figure 2) allows users to generated custom three-dimensional models of the budding yeast mitotic spindle and kinetochore complex. The program can model half of a mitotic spindle or an entire mitotic spindle by setting the “Number of Complexes” parameter to 1 or 2, respectively. The number and diameter of kinetochore microtubules can be set. The “Easy Align” setting forces the model to align to the *X*, *Y*, and *Z* axes of the visualization (bottom three panels) for easier interpretation of the model. The “Complex Diameter” parameter controls the diameter of the circular arrangement of kinetochore microtubules at their plus ends. The kinetochore microtubule plus ends can be randomly staggered by a given range, or can be staggered using a microtubule dynamics simulation using Simulink (Mathworks, Natick, MA, United States) (Stephens et al., 2013a). The “Rotation” parameter allows users to rotate the orientation of the plus ends of the kinetochore microtubules either randomly or by a fixed amount in the *X*, *Y*, and *Z* dimensions. The StaticKinet application was designed to simulate kinetochore protein distributions relative to the N-terminus of Nuf2. The N-terminus of Nuf2 is bound near the minus end of the kinetochore microtubule (Cheeseman et al., 2006; Wei et al., 2007; Ciferri et al., 2008). The “Length” parameter of Nuf2 sets the range the Nuf2 fluorophores will be distributed from the plus end of the microtubule. Thus, a “Length” parameter of 50 would distribute the Nuf2 fluorophores randomly in a 50 nm range from the plus end of the microtubule. The “Color Channel” parameter of Nuf2 controls the simulated color of the Nuf2 fluorophores. The StaticKinet application allows the user to control several parameters of the Spc29 distribution and the kinetochore microtubules. The Spc29 distribution allows users to approximate the structure of the spindle pole bodies (SPBs). The Spc29 parameters were designed so the user can either simulate the minus ends of the microtubules or adjust the model to create a disc-like structure by altering the structure and tubule diameters and tubule number parameters. The “Length” parameter controls the how widely the Spc29 fluorophores can distribute along the microtubule minus ends. The “Distance to (+)” parameter of Spc29 controls the average length of the kinetochore microtubules in the model, while the “Tubule Diameter” parameter of Spc29 controls the diameter of the minus ends of the kinetochore. The “Structure Diameter” parameter of Spc29 controls the diameter of the circular arrangement of the microtubule minus ends. The “Number of Fluorophore” parameter of Spc29 controls the number fluorophores per microtubule minus end. The “Number of Tubules” parameter of Spc29 controls the number of microtubule minus ends. The “Number of Tubules” parameter of Spc29 is independent of the “Number of Microtubules” parameter at the top of the interface that controls the number of microtubule plus ends. The “Color Channel” parameter of Spc29 controls the simulated color of the Spc29 fluorophores. The “Number of Arms” parameter under the kinetochore protein section controls the number of kinetochore fluorophores per microtubule plus end. The StaticKinet application models the kinetochores on a microtubule as straight rods coming from the plus ends of the microtubule to a single, defined point in space. The “Number of Bound Arms” controls the number of fluorophores simulated to be bound to a single point in space to mimic the individual kinetochore complexes converging on a single Cse4 centromere. Using the “Number of Bound Arms” parameter you can simulate a specified number of kinetochore complexes on each microtubule to randomly orient from the kinetochore plus end within a specified angle range. The length of the kinetochore fluorophore from the plus end is defined by the “Length of Arm” parameter. Users can slide the fluorophore along the simulated kinetochore “rod,” toward the microtubule plus end, by adjusting the “Point Marked” parameter. The “Angle from MT Axis” parameter controls the radial displacement of the single point in space where all the individual kinetochores converge, i.e., the Cse4 nucleosome. The parameter was meant to model the kinetochore complexes being pulled radially from the spindle. The “Help” tab allows users to access the online user guide. The “About” tab provides links to Bloom Lab code repository which contains the “KineticButShakeless” code repository^5^ that contains the StaticKinet application, the Bloom Laboratory website^6^, and the code webpage of the Bloom Lab website^7^. The “File” tab allows users to quit the application. The StaticKinetInMass application was used to generated multiple iterations of models designed in StaticsKinet. The StaticKinetInMass program outputted a series of XML files that contained the fluorophore locations that were converted into simulated microscope images by Microscope Simulator 2 (Quammen et al., 2008) available at http://cismm.web.unc.edu/software under the “Inactive Software” section.

### 3D Modeling and Simulated Imaging of the Budding Yeast Genome

The budding yeast genome was simulated as a polymer bead-chain model (Hult et al., 2017; Walker et al., 2019). Each chromosome was modeled as a polymer chain, with centromeres attached to a single point on the nuclear membrane. The genome was discretized such that each bead in the model composed approximately 5 kb of DNA. The model contained 2803 beads, of which 361 comprised the rDNA locus within the nucleolus. The beads representing the ribosomal DNA locus could spontaneously crosslink with each other to mimic the crosslinking effects of the structural maintenance of chromosome proteins, i.e. cohesin and condensin. The average duration of the crosslinks (μ) was set to either 0.09, 0.19, or 1.6 s in each simulation. The model was generated and simulated using DataTank, an object oriented programing environment^8^. DataTank’s microscope simulator module was used to generate simulated fluorescent images of the beads corresponding to the rDNA locus.

## Results

### A CNN Can Distinguish Fluorescent Images of GFP-Nuf2/SPC29-RFP and Cse4-GFP/SPC29-RFP

**FIGURE 3:**
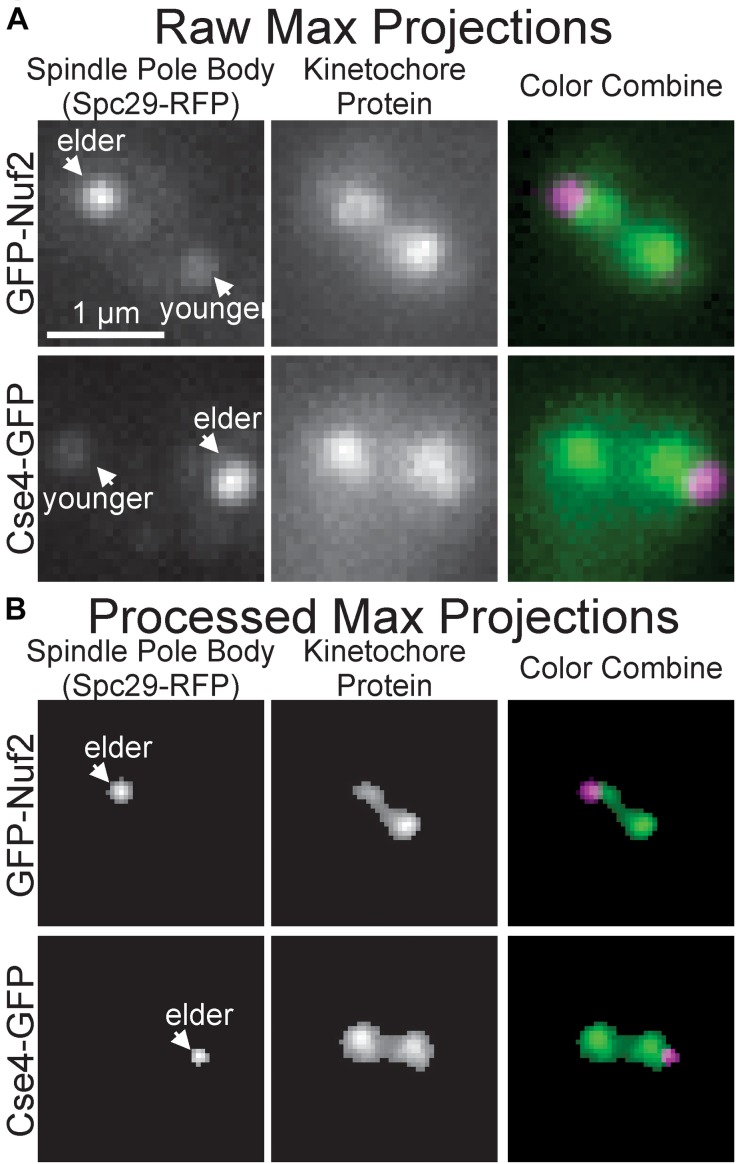
Representative raw and processed images of GFP-Nuf2 and Cse4-GFP. **(A)** Raw maximum intensity projections of N-terminal GFP-tagged Nuf2 and SPC29-RFP in mitotic budding yeast cell (upper row) and raw intensity projection of CSE4-GFP and SPC29 in mitotic budding yeast cell (lower row). Arrows indicate positions of elder and younger spindle pole body foci. **(B)** Processed versions of maximum intensity projections in **(A)**. Note loss of younger spindle pole body foci.

To initially determine whether the images of inner and outer kinetochore complexes are distinguishable, images of Spc29-RFP, GFP-Nuf2 (outer kinetochore) and Spc29-RFP, Cse4-GFP (inner kinetochore) were acquired through the automated image acquisition pipeline (see section “Experimental Image Acquisition, Segmentation, and Normalization”). The automated process of acquiring, segmenting, and preprocessing images of Spc29-RFP, GFP-Nuf2 and Spc29-RFP, Cse4-GFP generated 5,920 and 4,416 images, respectively (Figure 3). The elder SPB is brighter than the younger SPB when fluorescently tagged with RFP (Pereira et al., 2001). This mismatch in SPB foci brightness causes the younger SPB foci to occasionally be erased in during the image processing (Figures 3A,B). Of the 5,920 images of Nuf2, 4,416 were randomly selected to allow for a balanced dataset. The trained CNN was able to distinguish and categorize inner kinetochore images from outer kinetochore images at a 87.4% testing accuracy (Table 2), confirming that the Nuf2 and Cse4 processed images can be distinguished by a CNN.

**TABLE 2:** Confusion matrix of the CNN classification of processed, experimental images.

True classification	Cse4-GFP (*n* = 883)	754	129
	Nuf2-GFP (*n* = 883)	94	789
Total *N* = 1766		Cse4-GFP	Nuf2-GFP
		Predicted classification	

### Ranking Kinetochore Feature Importance With Principal Component Analysis

Previous studies have shown that the foci of Cse4-GFP in metaphase yeast are, on average, further from their proximal SPB foci parallel to the spindle (Haase et al., 2013) and are “taller” (wider perpendicular to the spindle) than outer kinetochore proteins (Haase et al., 2012). Therefore, we chose to measure both the Euclidian (the one-dimensional distance that combines the distances parallel and perpendicular to the SPB foci) distance and distance parallel to the spindle of kinetochore foci to their proximal SPB and the spot height (width perpendicular to the spindle). We reasoned that the distance between the Cse4-GFP foci would be greater than GFP-Nuf2 foci given Cse4-GFP foci appear further from their proximal SPB foci; however, this metric will also change as the cell cycle progresses so we wanted to query if the feature could still distinguish Cse4-GFP and GFP-Nuf2 images. Given that foci shape has been shown to be a distinguishing criterion for fluorescently tagged inner and outer kinetochore proteins, we wanted to examine the standard deviation of signal intensity of the foci both overall and parallel and perpendicular to the spindle. Previous measurements of foci intensity have shown that C-terminally tagged Nuf2-GFP is approximately 4-fold greater than Cse4-GFP (Joglekar et al., 2008a); therefore, we measured the mean intensity of the foci. The mother buds of mitotic yeast were automatically cropped, processed, and analyzed to extract features about the kinetochore foci’s shape, intensity and position relative to the SPB foci (see section “Experimental Image Acquisition, Segmentation, and Normalization”and “Feature Extraction and Principal-Component-Analysis-Based Feature Evaluation”). Images that lacked either kinetochore or SPB foci due to processing were disregarded by the feature extraction program. The resulting feature tables of Cse4-GFP and GFP-Nuf2 were joined and principal component analysis was performed to rank each feature’s importance (Table 1).

**FIGURE 4:**
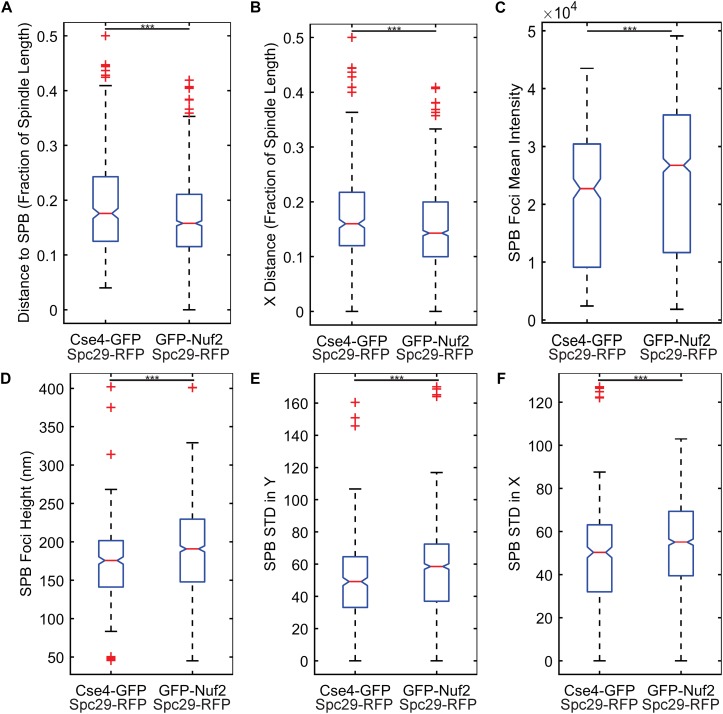
Feature comparisons between GFP-Nuf2 and Cse4-GFP processed images. Boxplots comparing kinetochore foci distance to proximal spindle pole body (SPB) foci **(A)**, kinetochore foci distance to proximal SPB foci in X-direction only (parallel to the spindle) **(B)**, SPB foci intensity **(C)**, SPB foci height (width of foci perpendicular to spindle) **(D)**, standard deviation of the intensities generated from a line-scan of a SPB foci perpendicular to the spindle **(E)**, standard deviation of the intensities generated from a line-scan of a SPB foci parallel to the spindle **(F)**. The three asterisks indicate a *p*-value < 0.001 using a Wilcoxon rank sum test.

The two most important features were kinetochore foci distance to the proximal SPB foci (kinetochore distance to SPB in Table 1) and kinetochore foci distance to the proximal SPB foci parallel to the spindle (kinetochore X-distance in Table 1), recapitulating the result from Haase et al. (2013) that inner kinetochore proteins are further from the proximal SPB than outer kinetochore proteins (Figures 4A,B). The next set of features all depended on the shape and intensity of the SPB foci. Statistical analysis of the processed images revealed that the GFP-Nuf2 strain’s SPB foci have higher mean intensities and have larger spot heights than SPB foci from the Cse4-GFP strain (Figures 4C–F). It is important to note that the images were rotated, rescaled, background subtracted, and de-noised using a custom MATLAB program (see section “Experimental Image Acquisition, Segmentation, and Normalization”). Therefore, the image processing enhanced differences in the appearance of SPB foci of the GFP-Nuf2 and Cse4-GFP strains. In contrast, the intensity of the kinetochore foci was one of the least important features (Table 1). This analysis was to aid in understanding what features the CNN may generate to distinguish processed images of GFP-Nuf2 and Cse4-GFP, as it is not possible to provide accurate quantitative measurements of fluorescent intensity from rescaled images.

**FIGURE 5:**
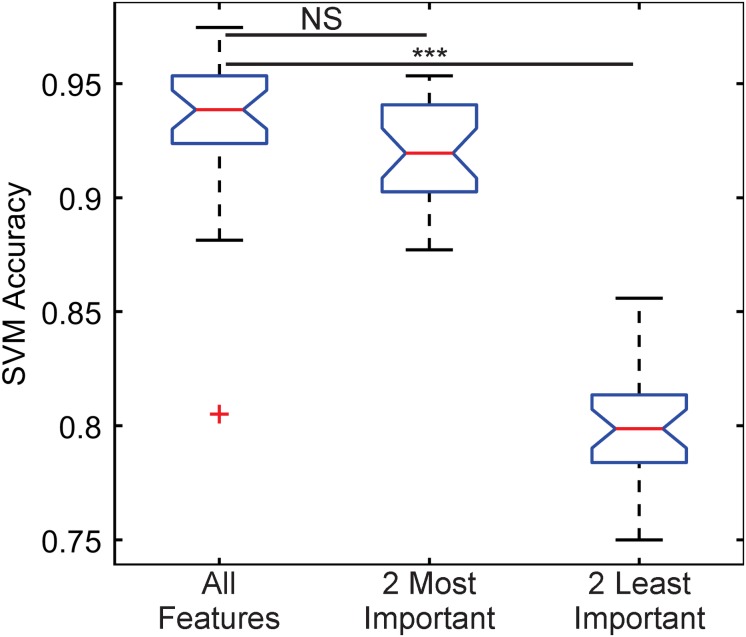
Boxplot comparing classification accuracies of differing sets of kinetochore image features. Thirty support vector machines (SVMs) were trained on randomly selected data composing 70% of the entire feature dataset, making each SVM independent. The accuracy of each SVM was calculated by having each trained SVM predict the classifications of the remaining 30% of the original dataset. The two most important features are kinetochore distance to SPB and kinetochore X-distance, and the two least important features are kinetochore foci mean intensity and kinetochore foci standard deviation in X-direction (see Table 1). Three asterisks indicate *P* < 0.001 using a Wilcoxon rank sum test. NS indicate *P* > 0.05 using a Wilcoxon rank sum test.

The importance of these features was tested by training three classification algorithms using a support vector machine (SVM) with a Gaussian kernel. One set would use all the features, another would use the two most important features, kinetochore distance to SPB and kinetochore X-distance (Table 1), and another would use the two least important features, kinetochore foci mean intensity and kinetochore foci standard deviation in X (parallel to spindle) (Table 1). For each set of features, the data was randomly split 70% for training and 30% for testing, and an SVM with a Gaussian kernel trained was trained and then predicted the classifications of the test set. To deduce the ability of each feature set to distinguish images of Cse4 and Nuf2, the data was randomly split into test and training sets 30 times and an SVM was trained and tested on the resulting sets each time to generate a distribution of testing accuracies. The accuracies of the SVM’s that were trained on the two most important features were not statistically different than the accuracies of the SVM’s trained with all the features (Figure 5). In contrast, the accuracies of the SVM’s that were trained on the two least important features were statistically lower than the accuracies of the SVM’s trained with all the features (Figure 5). Thus, PCA-based, exploratory data analysis showed that distance of the kinetochore foci to the proximal SPB foci, which had been observed to be different in a previous study (Haase et al., 2013), was an important, distinguishing feature of Cse4 and Nuf2 fluorescent images. This result shows that the PCA-based method can help users identify distinguishing features of differing biological structures.

### CNN-Based Approach to Forward Modeling of the Kinetochore

**FIGURE 6:**
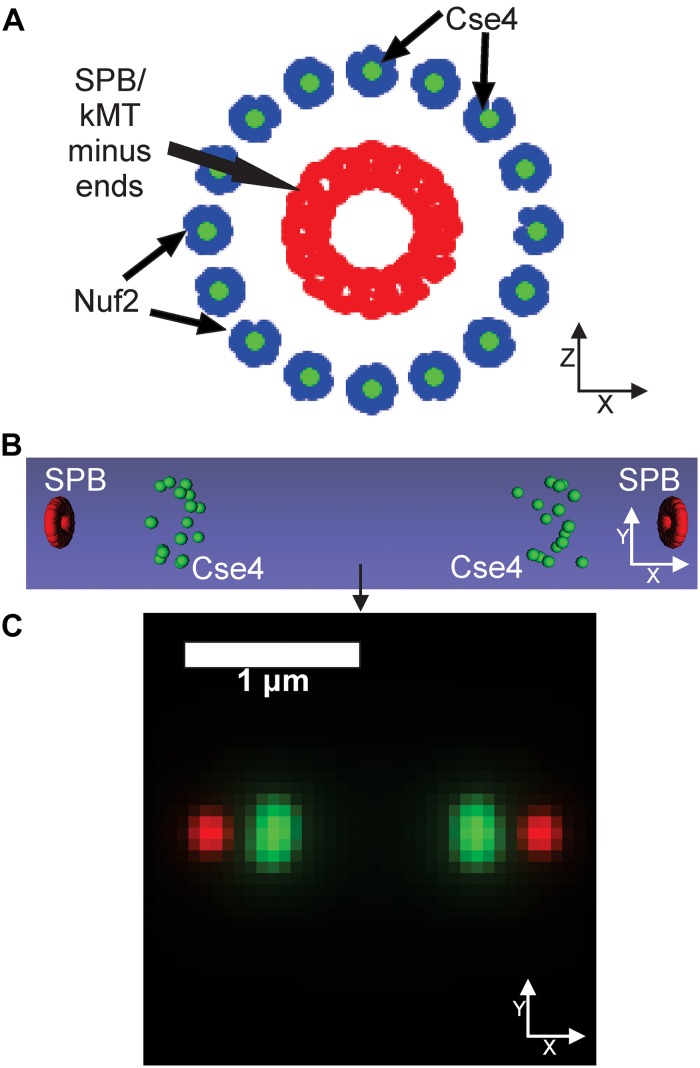
Default kinetochore model organization. **(A)** Cartoon of the kinetochore model. The model is oriented end-on from the spindle, so the sister kinetochore complexes and SPBs (red dots) overlay each other. Cse4s (green dots) are located on the central axis of the microtubule. The N-termini of Nuf2 are blue dots (Joglekar et al., 2009) that bind the plus-end of the simulated kinetochore microtubules (kMTs), which are not shown. SPBs (red dots) are represented in our model as fluorophores surrounding the minus ends of the kMTs. **(B)** Side-view of the volumetric model, generated by the Microscope Simulator 2 program, of the organization that will be used for simulated, fluorescent image generation. **(C)** The simulated microscope image of the organization shown in panel **(B)**.

Our PCA-based, exploratory data analysis relies on feature engineering, which requires a user to generate features themselves. On the other hand, a CNN requires no feature engineering but provides little information as to why the CNN classified images into a given category. We reasoned a three-dimensional computational model that could be converted into a simulated, fluorescent image could be used to train a CNN to provide users insights into what model parameters best fit experimental images. First, a suit of differing model parameters is established and for each parameter set, multiple simulated images are created and labeled to that parameter set. Those image sets are used to train a CNN. The trained CNN then classifies experimental images to a given parameter set. Users could then examine which model parameters consistently label experimental images. To test this approach, we wanted to explore how a CNN, trained on simulated images generated by a three-dimensional, kinetochore model, would classify experimental images of Cse4-GFP. Simulated images were developed based on our current understanding of the mitotic spindle and kinetochore. We based the dimensions of the mitotic spindle on electron tomographs of the budding yeast mitotic spindle (O’Toole et al., 1999). We built our model such that the N-terminus of Nuf2 (blue) binds to the plus-end of a kinetochore microtubule, based on the reports of the N-terminus of Nuf2 binding to kinetochore microtubules (Cheeseman et al., 2006; Wei et al., 2007; Ciferri et al., 2008). In the model, Cse4 (green) is placed in line with the central axis of the microtubule by default (Figure 6A), but the exact location of Cse4 relative to the attached kinetochore microtubule is unclear. In our model, the diameter of the spindle at the kinetochore plus ends was set to 250 nm, with each microtubule having a diameter of 25 nm (Sodeik, 2000), and the microtubule ends were staggered in a uniform distribution ± 100 nm from the central line, representing the range of microtubules lengths observed in bipolar spindles (O’Toole et al., 1999). We represented the SPB (red) by placing 20 fluorophores at the minus ends of the 16 kinetochore microtubules (Figure 6A), which is a rough approximation of the complex shape of the yeast SPB (Bullitt et al., 1997; Muller et al., 2005; Viswanath et al., 2017). Our computational model is converted to a fluorescent image using Microscope Simulator 2 (Quammen et al., 2008), which converts each Cse4 (green) and SPB (red) bead in Figure 6A into a single fluorophore positioned in three dimensions (Figure 6B) and then into a simulated, fluorescent image (Figure 6C) by convolving each fluorophore with a point-spread function. The spreading of the light via diffraction blurs the distributions of the fluorophores into individual foci (Figure 6C).

**FIGURE 7:**
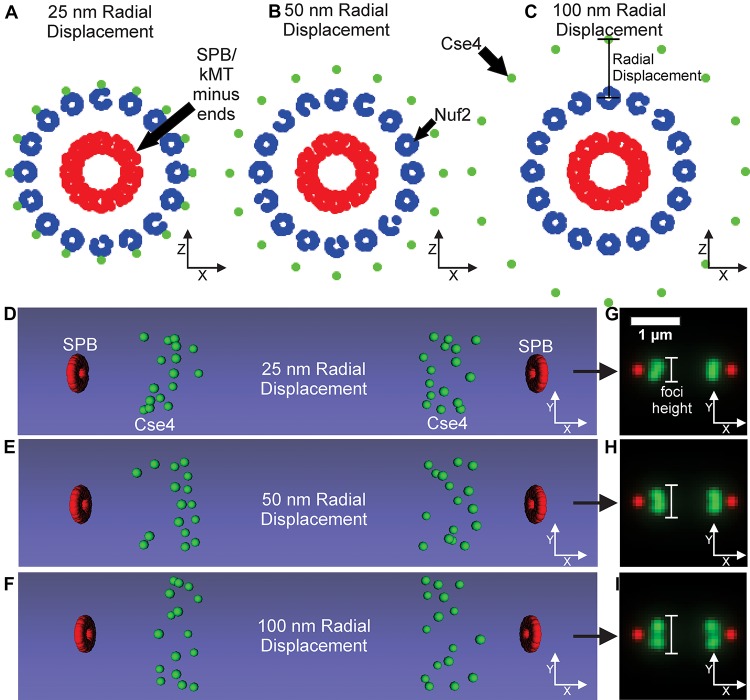
Kinetochore model organization with Cse4 radially displaced from kinetochore microtubule axis. **(A–C)** Scatter plots depicting the end-on view of the 25, 50, and 100 nm radial displacement models. Cse4s are the green dots, N-termini of Nuf2 are the blue dots forming a ring around the kMT plus-ends, and the SPBs are composed of red dots surrounding the minus-ends of the kMTs. **(D–F)** Volumetric models, generated by the Microscope Simulator 2 program, of the 25, 50, and 100 nm radial displacement organizations that were used for simulated, fluorescent image generation. **(G–I)** The respective simulated microscope images to the volumetric models shown in panels **(E,F)**. White bars indicate Cse4 foci height.

A controversial parameter in our model is the position and number of Cse4 molecules. The initial assumption in the field was that there was one Cse4-containing nucleosome (2 Cse4 proteins per nucleosome) per centromere in budding yeast (Meluh et al., 1998; Collins et al., 2004; Joglekar et al., 2008b). Using independent fluorescent standards, subsequent studies found the signal of metaphase Cse4 foci was brighter than expected (Coffman et al., 2011; Lawrimore et al., 2011) and that Cse4 foci were “taller” (wider perpendicular to the spindle) than outer kinetochore foci (Haase et al., 2012). These findings suggest that Cse4 proteins are spread perpendicular to the spindle in budding yeast during metaphase. Therefore, we altered the placement of Cse4 to gradually increase its radial distance from the corresponding kinetochore microtubule center in our computational model (Figures 7A–C). The radial models were converted into Microscope Simulator 2 simulations (Figures 7D–F) and then into simulated, fluorescent images (Figures 7G–I). The increase in radial displacement of Cse4 did result in noticeably “taller” Cse4 foci in the simulated images (Figures 7D–F). We trained a CNN on simulated images generated from models were Cse4 was displaced by 0 nm (Figure 6), 25 nm, 50 nm, or 100 nm (Figure 7). The validation accuracy of the CNN on simulated images was 99.98%, demonstrating the CNN could distinguish the change in Cse4 radial position with high accuracy (Table 3). We used the trained CNN to predict the radial displacement of 4,416 processed experimental images of Cse4-GFP and SPC29-RFP (Table 4). Most processed images were classified with a radial displacement of 25 or 50 nm, with only 1.9% (83 of 4,416) of images being classified as 0 nm. This result demonstrates that our CNN-based approach to forward modeling captured the biological observation that Cse4-GFP foci are spread perpendicular to the spindle axis. Thus, this CNN-based approach to forward modeling is suitable for fine tuning computational models of biological structures.

**TABLE 3:** Confusion matrix of the CNN classification of simulated Cse4 images of varying radial displacements.

True classification	Sim. 0 nm/On-axis (*n* = 14,987)	14,984	3	0	0
	Sim. 25 nm (*n* = 14,832)	0	14,825	7	0
	Sim. 50 nm (*n* = 14,706)	0	2	14,703	1
	Sim. 100 nm (*n* = 14,130)	0	0	0	14,130
Total *N* = 58,655		Sim. 0 nm/On-axis	Sim. 25 nm	Sim. 50 nm	Sim. 100 nm
		Predicted classification			

**TABLE 4:** Categorization of experimentally acquired inner kinetochore images by the CNN trained on simulated images of the inner kinetochore of varying radial displacements.

Exp. Cse4	83	2954	1379	0
Total *N* = 4,416	Sim. 0 nm/On-axis	Sim. 25 nm	Sim 50 nm	Sim 100 nm
	Predicted classification			

### CNN Classification of Simulated Nucleolar Images

**FIGURE 8:**
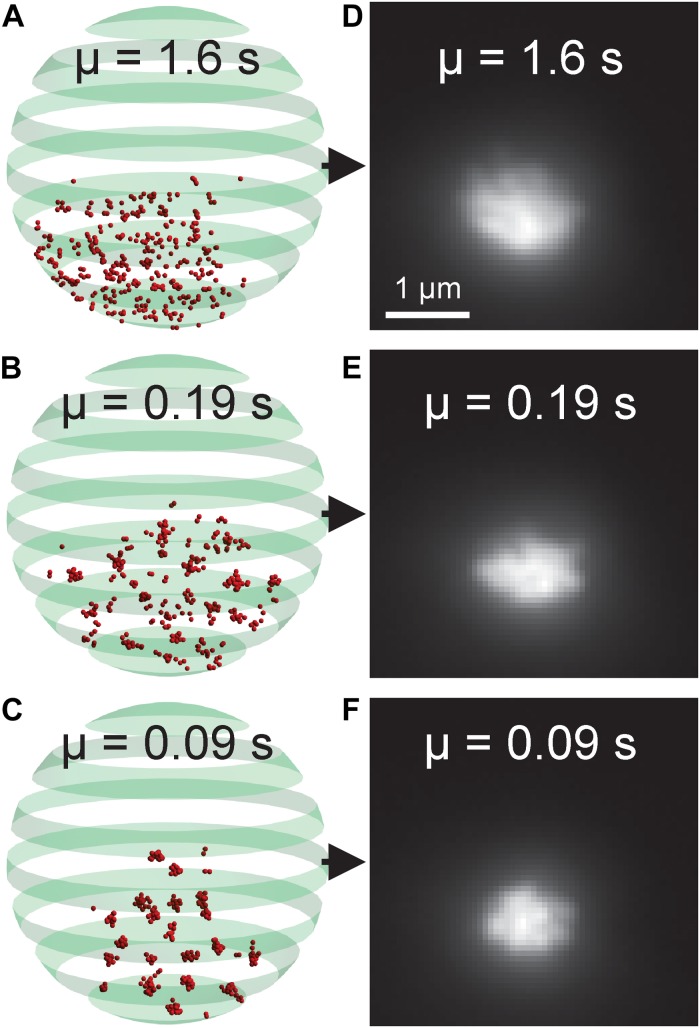
Nucleolar model organization and simulated fluorescent images. **(A–C)** Volumetric model of the budding yeast nucleolus with differing bead-bead dynamic crosslinking durations. Red beads represent rDNA locus. The green banded sphere represents the nuclear membrane which acts as a boundary constraint in the simulation. **(D–F)** Simulated fluorescent images of the nucleolar models. The parameter μ indicates the average duration of a bead-bead crosslink.

The nucleolus is the region within the nucleus that surrounds the rDNA locus. Fluorescently labeled nucleolar proteins appear amorphous, making identification of morphological phenotypes difficult. Recently, two studies utilized three-dimensional, computational simulations of the budding yeast genome to study how different DNA crosslinking kinetics within the rDNA locus affect nucleolar sequestration and rDNA structure (Hult et al., 2017; Walker et al., 2019). We reasoned our CNN-based, forward modeling approach would be appropriate for this computational model if a CNN could detect the subtle differences in the simulated images generated from models with different crosslinking kinetics (Figure 8). We found that the CNN was able to identify the average crosslinking duration with 99.76% accuracy (Table 5). Thus, this rDNA model and CNN architecture would be suitable for CNN-based forward modeling.

**TABLE 5:** Confusion matrix of the CNN trained on simulated nucleolar images with varying crosslinking durations within the rDNA locus.

True classification	μ = 1.6 s (*n* = 2,088)	2,086	2	0
	μ = 0.19 s (*n* = 2,088)	7	2,081	0
	μ = 0.09 s (*n* = 2,088)	0	6	2,082
Total *N* = 6,264		μ = 1.6 s	μ = 0.19 s	μ = 0.09 s
		Predicted classification		

## Discussion

### The Kinetochore as a Test-Case for AI-Assisted, Forward Modeling

Here we demonstrate a method that combines deep learning and computational modeling to compare models of biological structures directly with experimental images (Figure 1). A relatively simple CNN, built with MATLAB’s Deep Learning ToolBox (Mathworks, Natick, MA, United States), was able to determine the difference between GFP-Nuf2/Spc29-RFP image and Cse4-GFP/Spc29-RFP images with 87.4% accuracy (Table 2). To validate the difference between the images of GFP-Nuf2/Spc29-RFP and Cse4-GFP/Spc29-RFP, we extracted features that were known to be different between the two strains. The importance of these features was determined using PCA and validated by comparing the classification accuracies of SVM’s trained on features deemed important versus SVM’s trained on features deemed unimportant. Indeed, the SVM’s trained on unimportant feature performed worse than those trained on important features (Figure 5). The most important feature was the distance of the kinetochore foci to the proximal SPB foci (Table 1), that had previously been shown to be different between Cse4 and Nuf2 (Haase et al., 2013). Thus, this PCA-based exploratory data analysis can detect biologically relevant difference between fluorescent images. However, the PCA-based analysis can only indicate a feature as discernable. The PCA-based analysis cannot discern the appropriate value of a given feature. In order to determine the measurements of features we generated a three-dimensional, computational model of the kinetochore. We then altered the radial displacement of Cse4 in the model to determine which radial displacement settings fit the experimental images. The CNN classified 98.1% of experimental images of Cse4-GFP in the 25 nm and 50 nm radial displacement categories (Table 4). This result recapitulated the biological finding that Cse4-GFP is “taller” (wider perpendicular to the spindle) than other fluorescently labeled outer kinetochore proteins (Haase et al., 2012).

The kinetochore model illustrated that a CNN used in conjunction with a three-dimensional model can be used to fine-tune model parameters to match experimental images. We used the kinetochore as test case for our AI-assisted forward modeling pipeline, but any biological structure that can be imaged and computationally modeled can be analyzed by this method. Unlike the kinetochore, images of fluorescently labeled nucleolar proteins often appear amorphous, making CNN-based forward modeling particularly useful. We again found that a relatively simple CNN, built with MATLAB’s Deep Learning ToolBox (Mathworks, Natick, MA, United States), was able to determine the difference between simulated nucleolar images (Figure 8) generated from a budding yeast genome model (Hult et al., 2017; Walker et al., 2019) with 99.76% accuracy (Table 5). Thus, a CNN is capable of discerning differences in amorphous structures as well.

### Future Directions for AI-Assisted Forward Modeling

Here, we only altered a single parameter, the radial displacement of Cse4, in our computational model. However, it is possible to alter multiple parameters of our model and train a CNN on the different combinations allowing us to fine tune the model on multiple parameters. An alternative approach is to use a system similar to a generative adversarial network (GAN) (Goodfellow et al., 2014). GANs have been used recently to generate synthetic super-resolution images from lower resolution microscopy images (Ouyang et al., 2018; Wang et al., 2019; Zhang et al., 2019). In this approach the computational model would be driven by a generative algorithm to generate simulated images. A discriminative algorithm that had been trained on experimental images would then classify the simulated image into a given class or flag it as a detected fake. Since each simulated image is derived from a model, the generative algorithm could automatically tune model parameters to best fit an experimental image dataset. The generated images could them be interrogated by feature extraction to ensure key feature metrics were maintained in the simulated image generation. This GAN-based approach is a natural extension of the method we have described here and could be used on any biological structure that can be imaged and computationally modeled.

## Data Availability Statement

The datasets generated for this study are available on request to the corresponding author.

## Author Contributions

JL provided technical guidance for the computer simulations and construction of the analysis pipeline, implemented the analysis, and wrote the manuscript. AD built the kinetochore simulation and analysis pipeline, generated the implemented the analysis, and wrote the manuscript. BW constructed and ran the budding yeast genome simulations and generated the simulated images of fluorescently labeled rDNA loci. KB and JL provided the biological framework and assisted with the completion of the manuscript.

## Conflict of Interest

The authors declare that the research was conducted in the absence of any commercial or financial relationships that could be construed as a potential conflict of interest.

## Footnotes

1 https://github.com/jlaw8504/CellStarSelect

2 https://github.com/HMS-IDAC/MatBots

3 https://github.com/BloomLabYeast/CNNs

4 https://github.com/BloomLabYeast/FeatureExtractionAndSVM

5 https://github.com/BloomLabYeast/KineticButShakeless

6 http://bloomlab.web.unc.edu/

7 http://bloomlab.web.unc.edu/resources/programs/

8 http://www.visualdatatools.com/DataTank

## References

[B1] Aguet F. Antonescu C. N. Mettlen M. Schmid S. L. Danuser G. (2013). Advances in analysis of low signal-to-noise images link dynamin and AP2 to the functions of an endocytic checkpoint. *Dev. Cell* 26 279–291. 10.1016/j.devcel.2013.06.019 23891661 PMC3939604

[B2] Alushin G. Nogales E. (2011). Visualizing kinetochore architecture. *Curr. Opin. Struct. Biol.* 21 661–669. 10.1016/j.sbi.2011.07.009 21862320 PMC3189262

[B3] Anedchenko E. A. Samel-Pommerencke A. Tran Nguyen T. M. Shahnejat-Bushehri S. Popsel J. Lauster D. (2019). The kinetochore module Okp1(CENP-Q)/Ame1(CENP-U) is a reader for N-terminal modifications on the centromeric histone Cse4(CENP-A). *EMBO J.* 38:e98991. 10.15252/embj.201898991 30389668 PMC6315295

[B4] Biggins S. (2013). The composition, functions, and regulation of the budding yeast kinetochore. *Genetics* 194 817–846. 10.1534/genetics.112.145276 23908374 PMC3730914

[B5] Bullitt E. Rout M. P. Kilmartin J. V. Akey C. W. (1997). The yeast spindle pole body is assembled around a central crystal of Spc42p. *Cell* 89 1077–1086. 10.1016/s0092-8674(00)80295-0 9215630

[B6] Cheeseman I. M. Chappie J. S. Wilson-Kubalek E. M. Desai A. (2006). The conserved KMN network constitutes the core microtubule-binding site of the kinetochore. *Cell* 127 983–997. 10.1016/j.cell.2006.09.039 17129783

[B7] Cicconet M. Hochbaum D. Richmond D. Sabatin B. (2017). “Bots for software-assisted analysis of image-based transcriptomics,” in *Proceedings of theIEEE International Conference on Computer Vision Workshop (ICCVW)*, (Venice: IEEE), 134–142.

[B8] Ciferri C. Pasqualato S. Screpanti E. Varetti G. Santaguida S. Dos Reis G. (2008). Implications for kinetochore-microtubule attachment from the structure of an engineered Ndc80 complex. *Cell* 133 427–439. 10.1016/j.cell.2008.03.020 18455984 PMC4754795

[B9] Coffman V. C. Wu P. Parthun M. R. Wu J. Q. (2011). CENP-A exceeds microtubule attachment sites in centromere clusters of both budding and fission yeast. *J. Cell Biol.* 195 563–572. 10.1083/jcb.201106078 22084306 PMC3257534

[B10] Collins K. A. Furuyama S. Biggins S. (2004). Proteolysis contributes to the exclusive centromere localization of the yeast Cse4/CENP-A histone H3 variant. *Curr. Biol.* 14 1968–1972. 10.1016/j.cub.2004.10.024 15530401

[B11] Fukagawa T. Earnshaw W. C. (2014). The centromere: chromatin foundation for the kinetochore machinery. *Dev. Cell* 30 496–508. 10.1016/j.devcel.2014.08.016 25203206 PMC4160344

[B12] Gardner M. K. Bouck D. C. Paliulis L. V. Meehl J. B. O’toole E. T. Haase J. (2008). Chromosome congression by Kinesin-5 motor-mediated disassembly of longer kinetochore microtubules. *Cell* 135 894–906. 10.1016/j.cell.2008.09.046 19041752 PMC2683758

[B13] Goodfellow I. J. Pouget-Abadie J. Mirza M. Xu B. Warde-Farley D. Ozair S. (2014). “Generative adversarial networks,” in *Proceedings of the 27th International Conference on Neural Information Processing Systems*, Montreal.

[B14] Haase J. Mishra P. K. Stephens A. Haggerty R. Quammen C. Taylor R. M. (2013). A 3D map of the yeast kinetochore reveals the presence of core and accessory centromere-specific histone. *Curr. Biol.* 23 1939–1944. 10.1016/j.cub.2013.07.083 24076245 PMC3796065

[B15] Haase J. Stephens A. Verdaasdonk J. Yeh E. Bloom K. (2012). Bub1 kinase and Sgo1 modulate pericentric chromatin in response to altered microtubule dynamics. *Curr. Biol.* 22 471–481. 10.1016/j.cub.2012.02.006 22365852 PMC3311747

[B16] He X. Rines D. R. Espelin C. W. Sorger P. K. (2001). Molecular analysis of kinetochore-microtubule attachment in budding yeast. *Cell* 106 195–206. 10.1016/s0092-8674(01)00438-x 11511347

[B17] Hornung P. Troc P. Malvezzi F. Maier M. Demianova Z. Zimniak T. (2014). A cooperative mechanism drives budding yeast kinetochore assembly downstream of CENP-A. *J. Cell Biol.* 206 509–524. 10.1083/jcb.201403081 25135934 PMC4137059

[B18] Huis in ’t Veld P. J. Jeganathan S. Petrovic A. Singh P. John J. Krenn V. (2016). Molecular basis of outer kinetochore assembly on CENP-T. *eLife* 5:e21007. 10.7554/eLife.21007 28012276 PMC5241120

[B19] Hult C. Adalsteinsson D. Vasquez P. A. Lawrimore J. Bennett M. York A. (2017). Enrichment of dynamic chromosomal crosslinks drive phase separation of the nucleolus. *Nucleic Acids Res.* 45 11159–11173. 10.1093/nar/gkx741 28977453 PMC5737219

[B20] Joglekar A. P. (2016). A cell biological perspective on past, present and future investigations of the spindle assembly checkpoint. *Biology* 5:E44. 27869759 10.3390/biology5040044 PMC5192424

[B21] Joglekar A. P. Bloom K. Salmon E. D. (2009). In vivo protein architecture of the eukaryotic kinetochore with nanometer scale accuracy. *Curr. Biol.* 19 694–699. 10.1016/j.cub.2009.02.056 19345105 PMC2832475

[B22] Joglekar A. P. Bouck D. Finley K. Liu X. Wan Y. Berman J. (2008a). Molecular architecture of the kinetochore-microtubule attachment site is conserved between point and regional centromeres. *J. Cell Biol.* 181 587–594. 10.1083/jcb.200803027 18474626 PMC2386099

[B23] Joglekar A. P. Salmon E. D. Bloom K. S. (2008b). Counting kinetochore protein numbers in budding yeast using genetically encoded fluorescent proteins. *Methods Cell Biol.* 85 127–151. 10.1016/s0091-679x(08)85007-8 18155462 PMC2892121

[B24] Joglekar A. P. Kukreja A. A. (2017). How kinetochore architecture shapes the mechanisms of its function. *Curr. Biol.* 27 R816–R824. 10.1016/j.cub.2017.06.012 28829971 PMC5721348

[B25] Kitagawa K. Hieter P. (2001). Evolutionary conservation between budding yeast and human kinetochores. *Nat. Rev. Mol. Cell Biol.* 2 678–687. 10.1038/35089568 11533725

[B26] Kraus O. Z. Ba J. L. Frey B. J. (2016). Classifying and segmenting microscopy images with deep multiple instance learning. *Bioinformatics* 32 i52–i59. 10.1093/bioinformatics/btw252 27307644 PMC4908336

[B27] Lampert F. Westermann S. (2011). A blueprint for kinetochores - new insights into the molecular mechanics of cell division. *Nat. Rev. Mol. Cell Biol.* 12 407–412. 10.1038/nrm3133 21633384

[B28] Lara-Gonzalez P. Westhorpe F. G. Taylor S. S. (2012). The spindle assembly checkpoint. *Curr. Biol.* 22 R966–R980. 10.1016/j.cub.2012.10.006 23174302

[B29] Lawrimore J. Aicher J. K. Hahn P. Fulp A. Kompa B. Vicci L. (2016). ChromoShake: a chromosome dynamics simulator reveals that chromatin loops stiffen centromeric chromatin. *Mol. Biol. Cell* 27 153–166. 10.1091/mbc.E15-08-0575 26538024 PMC4694754

[B30] Lawrimore J. Bloom K. S. Salmon E. D. (2011). Point centromeres contain more than a single centromere-specific Cse4 (CENP-A) nucleosome. *J. Cell Biol.* 195 573–582. 10.1083/jcb.201106036 22084307 PMC3257525

[B31] LeCun Y. Bengio Y. Hinton G. (2015). Deep learning. *Nature* 521:436. 10.1038/nature14539 26017442

[B32] Léger-Silvestre I. Trumtel S. Noaillac-Depeyre J. Gas N. (1999). Functional compartmentalization of the nucleus in the budding yeast Saccharomyces cerevisiae. *Chromosoma* 108 103–113. 10.1007/s004120050357 10382072

[B33] Lu A. X. Zarin T. Hsu I. S. Moses A. M. (2019). YeastSpotter: accurate and parameter-free web segmentation for microscopy images of yeast cells. *Bioinformatics* 35 4525–4527. 10.1093/bioinformatics/btz402 31095270 PMC6821424

[B34] Meluh P. B. Yang P. Glowczewski L. Koshland D. Smith M. M. (1998). Cse4p is a component of the core centromere of Saccharomyces cerevisiae. *Cell* 94 607–613. 10.1016/s0092-8674(00)81602-5 9741625

[B35] Muller E. G. D. Snydsman B. E. Novik I. Hailey D. W. Gestaut D. R. Niemann C. A. (2005). The organization of the core proteins of the yeast spindle pole body. *Mol. Biol. Cell* 16 3341–3352. 10.1091/mbc.e05-03-0214 15872084 PMC1165416

[B36] Musacchio A. Desai A. (2017). A molecular view of kinetochore assembly and function. *Biology* 6:E5. 10.3390/biology6010005 28125021 PMC5371998

[B37] O’Toole E. T. Winey M. Mcintosh J. R. (1999). High-voltage electron tomography of spindle pole bodies and early mitotic spindles in the yeast Saccharomyces cerevisiae. *Mol. Biol. Cell* 10 2017–2031. 10.1091/mbc.10.6.2017 10359612 PMC25406

[B38] Ouyang W. Aristov A. Lelek M. Hao X. Zimmer C. (2018). Deep learning massively accelerates super-resolution localization microscopy. *Nat. Biotechnol.* 36:460. 10.1038/nbt.4106 29658943

[B39] Pärnamaa T. Parts L. (2017). Accurate classification of protein subcellular localization from high-throughput microscopy images using deep learning. *G3* 7 1385–1392. 10.1534/g3.116.033654 28391243 PMC5427497

[B40] Pereira G. Tanaka T. U. Nasmyth K. Schiebel E. (2001). Modes of spindle pole body inheritance and segregation of the Bfa1p–Bub2p checkpoint protein complex. *EMBO J.* 20 6359–6370. 10.1093/emboj/20.22.6359 11707407 PMC125717

[B41] Peterson J. B. Ris H. (1976). Electron-microscopic study of the spindle and chromosome movement in the yeast Saccharomyces cerevisiae. *J. Cell Sci.* 22 219–242. 794073 10.1242/jcs.22.2.219

[B42] Pot I. Knockleby J. Aneliunas V. Nguyen T. Ah-Kye S. Liszt G. (2005). Spindle checkpoint maintenance requires Ame1 and Okp1. *Cell Cycle* 4 1448–1456. 10.4161/cc.4.10.2106 16177574

[B43] Quammen C. W. Richardson A. C. Haase J. Harrison B. D. Taylor R. M. Bloom K. S. (2008). FluoroSim: a visual problem-solving environment for fluorescence microscopy. *Eurogr. Workshop Vis. Comput. Biomed.* 2008 151–158. 20431698 10.2312/VCBM/VCBM08/151-158 PMC2860625

[B44] Schmitzberger F. Richter M. M. Gordiyenko Y. Robinson C. V. Dadlez M. Westermann S. (2017). Molecular basis for inner kinetochore configuration through RWD domain-peptide interactions. *EMBO J.* 36 3458–3482. 10.15252/embj.201796636 29046335 PMC5709738

[B45] Sodeik B. (2000). Mechanisms of viral transport in the cytoplasm. *Trends Microbiol.* 8 465–472. 10.1016/s0966-842x(00)01824-2 11044681

[B46] Stephens A. D. Haggerty R. A. Vasquez P. A. Vicci L. Snider C. E. Shi F. (2013a). Pericentric chromatin loops function as a nonlinear spring in mitotic force balance. *J. Cell Biol.* 200 757–772. 10.1083/jcb.201208163 23509068 PMC3601350

[B47] Stephens A. D. Quammen C. W. Chang B. Haase J. Taylor R. M. II Bloom K. (2013b). The spatial segregation of pericentric cohesin and condensin in the mitotic spindle. *Mol. Biol. Cell* 24 3909–3919. 10.1091/mbc.E13-06-0325 24152737 PMC3861086

[B48] Stoler S. Keith K. C. Curnick K. E. Fitzgerald-Hayes M. (1995). A mutation in CSE4, an essential gene encoding a novel chromatin-associated protein in yeast, causes chromosome nondisjunction and cell cycle arrest at mitosis. *Genes Dev.* 9 573–586. 10.1101/gad.9.5.573 7698647

[B49] Sullivan D. P. Winsnes C. F. Åkesson L. Hjelmare M. Wiking M. Schutten R. (2018). Deep learning is combined with massive-scale citizen science to improve large-scale image classification. *Nat. Biotechnol.* 36 820–828. 10.1038/nbt.4225 30125267

[B50] Versari C. Stoma S. Batmanov K. Llamosi A. Mroz F. Kaczmarek A. (2017). Long-term tracking of budding yeast cells in brightfield microscopy: cell star and the evaluation platform. *J. R. Soc. Interface* 14:20160705. 10.1098/rsif.2016.0705 28179544 PMC5332563

[B51] Viswanath S. Bonomi M. Kim S. J. Klenchin V. A. Taylor K. C. Yabut K. C. (2017). The molecular architecture of the yeast spindle pole body core determined by Bayesian integrative modeling. *Mol. Biol. Cell* 28 3298–3314. 10.1091/mbc.E17-06-0397 28814505 PMC5687031

[B52] Walker B. Taylor D. Lawrimore J. Hult C. Adalsteinsson D. Bloom K. (2019). Transient crosslinking kinetics optimize gene cluster interactions. *PLoS Comput. Biol.* 15:e1007124. 10.1371/journal.pcbi.1007124 31433796 PMC6730938

[B53] Wang H. Rivenson Y. Jin Y. Wei Z. Gao R. Gunaydin H. (2019). “Cross-modality super-resolution in fluorescence microscopy enabled by generative adversarial networks,” in *Proceedings of the BMES (Biomedical Engineering Society) Annual Meeting*, Philadelphia.

[B54] Wei R. R. Al-Bassam J. Harrison S. C. (2007). The Ndc80/HEC1 complex is a contact point for kinetochore-microtubule attachment. *Nat. Struct. Mol. Biol.* 14 54–59. 17195848 10.1038/nsmb1186

[B55] Wong H. Marie-Nelly H. Herbert S. Carrivain P. Blanc H. Koszul R. (2012). A predictive computational model of the dynamic 3D interphase yeast nucleus. *Curr. Biol.* 22 1881–1890. 10.1016/j.cub.2012.07.069 22940469

[B56] Yang C. H. Lambie E. J. Hardin J. Craft J. Snyder M. (1989). Higher order structure is present in the yeast nucleus: autoantibody probes demonstrate that the nucleolus lies opposite the spindle pole body. *Chromosoma* 98 123–128. 2673672 10.1007/BF00291048

[B57] Zhang H. Fang C. Xie X. Yang Y. Mei W. Jin D. (2019). High-throughput, high-resolution deep learning microscopy based on registration-free generative adversarial network. *Biomed. Opt. Exp.* 10 1044–1063. 10.1364/BOE.10.001044 30891329 PMC6420277

